# Out-of-Home Food Consumers in Brazil: What Do They Eat?

**DOI:** 10.3390/nu10020218

**Published:** 2018-02-16

**Authors:** Giovanna Calixto Andrade, Maria Laura da Costa Louzada, Catarina Machado Azeredo, Camila Zancheta Ricardo, Ana Paula Bortolleto Martins, Renata Bertazzi Levy

**Affiliations:** 1 Núcleo de Pesquisas Epidemiológicas em Nutrição e Saúde (NUPENS), São Paulo 01246-907, Brazil; maria.laura.louzada@gmail.com (M.L.d.C.L.); catarina.azeredo@yahoo.com.br (C.M.A.); ca.zancheta@gmail.com (C.Z.R.); anapbmartins@gmail.com (A.P.B.M.); rlevy@usp.br (R.B.L.); 2 Departamento de Medicina Preventiva da, Faculdade de Medicina da, Universidade de São Paulo (FMUSP), São Paulo 01246-903, Brazil; 3 Departamento de Políticas Públicas e Saúde Coletiva, Universidade Federal de São Paulo (UNIFESP), São Paulo 11015-020, Brazil; 4 Curso de Nutrição da, Faculdade de Medicina da, Universidade Federal de Uberlândia (UFU), Uberlândia 38400-902, Brazil; 5 Instituto de Defesa do Consumidor (IDEC), São Paulo 05002-000, Brazil

**Keywords:** out-of-home eating, ultra-processed food, food patterns

## Abstract

Considering the increased contribution of foods consumed outside home and their potential impact on diet, this study aims to identify eating out patterns and their association with nutritional dietary quality in Brazil. We used the Individual Food Intake Survey 2008–2009, conducted with 34,003 individuals aged 10 and up. We used factor analysis by principal component to identify out-of-home eating patterns and linear regression to explore the association between patterns scores and dietary quality. We identified three food patterns. The “Traditional meal” pattern carried more rice, beans, meat, roots and tubers, pasta, vegetables and eggs. The “typical Brazilian breakfast/tea” pattern carried more fresh bread, margarine, milk, cheese and butter. The “Ultra-processed food” pattern carried more ready-to-eat meals and soft drinks. The “traditional meal” pattern was positively associated with calories from proteins, fiber, iron, potassium and sodium densities, whereas “typical Brazilian breakfast/tea” and “ultra-processed food” patterns were positively associated with energy density, the percentage of calories from lipids or carbohydrates, *trans* fat and free sugar. Out-of-home eating may have a negative impact on nutritional dietary quality when based on ultra-processed food. However, it is possible to maintain a healthy out-of-home diet with adherence to traditional Brazilian cuisine.

## 1. Introduction

Chronic non-communicable diseases (NCDs) are the world’s main causes of death, especially in low and middle-income countries, accounting for 59.7% of total global deaths in 2015 and 71% of total Brazilian deaths [1]. The increase of chronic NCDs such as diabetes, chronic respiratory diseases, cardiovascular disease and cancer are attributed in part to changes in dietary and lifestyle patterns [2].

Among dietary pattern changes, it is important to underscore the increased prevalence of out-of-home eating as a habit deemed “convenient” in terms of reduced demands in cleaning and preparation time, as well as its portability [3]. In high-income countries such as the United States, food eaten out-of-home is associated with low fiber, calcium and iron content and high amounts of saturated fat, cholesterol and sodium [3,4], high energetic density and high processing level [3,5,6]. Furthermore, the habit of eating out favors the substitution of traditional meals by snacks and fast-food [3,5,6] and it is associated with the consumption of larger food portions (encouraging people to eat more than necessary) [7].

In Brazil, national surveys show the increasing percentage of calories spent on foods eaten out of home from 24% to 31% of the total diet between 2002–2003 and 2008–2009 [8,9]. Out-of-home eating is associated with the consumption of unhealthy food items such as soda, fried appetizers, sweets and fast food [10,11].

Although previous studies indicated an association between out-of-home eating and low dietary quality in Brazil, they address the consumption of isolated food items or nutrients [10,11], which not necessarily reflects the interaction between foods and nutrients nor either an overall dietary pattern. A broad understanding of the cultural practices with regard to out-of-home eating depends on analyses of what foods tend to converge within a diet pattern or within a meal, since is the total diet and not one food item or nutrient that should be classified as healthy or unhealthy [4].

Therefore, the present study aims to identify out-of-home eating patterns in the Brazilian population and to examine its relationship with sociodemographic characteristics and the overall nutritional quality of the Brazilian diet.

## 2. Methods

### 2.1. Design and Population

We performed a cross-sectional analysis using the Individual Food Intake Survey collected as part of the Brazilian Institute of Geography and Statistics (IBGE) National Household Budget Survey (POF), conducted between May 2008 and May 2009 in Brazil.

The survey used a complex clustered sampling procedure, first selecting census areas and then selecting households within those areas. The selection of census areas was preceded by an examination of the areas of the Master Sample of Household Surveys or Common Sample to obtain strata of households with high geographic and socioeconomic homogeneity. Among the 55,970 households selected by the POF, a subsample consisting of 13,569 households (24.5%) was randomly selected. All individuals aged ≥10 years in the selected households were included in the survey and completed the Individual Food Intake Survey module. In this module, they completed two non-consecutive 24 h food records on predetermined days spanning one week and food consumption was estimated through the average of the two food records yielding a sample of 34,003 individuals.

Were excluded individuals who did not complete both days of the food record (3.2% of the population). As the study focus was evaluating out of home eaten, individuals who did not report eat at least one meal away from home on one of the two days of food record were not included in the analyses (52.9%). The final sample was 14,925.

Ethical approval was obtained by the Research Ethics Committee of the Faculdade de Medicina da Universidade de São Paulo (n^o^. 266/15).

### 2.2. Food Consumption

Food consumption was measured through food records self-reported by the participants or with the assistance of another resident (when, for some reason, it was impossible to do it alone). Each individual received a material guiding the way of filling the records, containing photos of home measures to assist in the estimation of quantities. The participants were instructed to log all food and beverages (except water) consumed in a 24-h period, including preparation, amount, time and place of the meal (inside or outside home). A question about the habit of sweetening drinks with sugar or sweetener was also included [12].

In the end, the agents reviewed the records, investigating the consumption of commonly forgotten foods and making corrections to the participants when necessary, eventually transcribing information into the electronic system. As part of IBGE quality control, food records with apparent collection errors were imputed. For more information on quality control procedures see Pesquisa de orçamentos familiares 2008–2009: análise do consumo alimentar pessoal no Brasil, IBGE, 2011 [12].

Reported food and beverage amounts were converted into grams and milliliters, respectively, based on food portion table [13]. Dietary energy and nutrients intakes we estimated based on Brazilian food composition table [14]. Following the survey guidelines, for the habit of sweetening drinks, when adding only sugar to beverages, 10.0% of the consumed volume of fruit juices, coffee and tea is to be considered as sugar content and when reporting a habit of adding sugar and sweeteners this value drops to 5% [14].

IBGE defines food eaten outside of the home as “all food items that were acquired and consumed away from home,” not including eating home-made food or take-out food outside of home.

All 1120 food items were categorized according to NOVA classification, which is based on the extent and purpose of industrial food processing. NOVA (not an acronym) divides food items into four groups: unprocessed or minimally processed foods, processed culinary ingredients, processed food and ultra-processed foods and drink products [15,16].

Unprocessed or minimally processed food group is obtained from plants or animals in nature, or with minimal alteration (such as cleaning, removal of inedible or unwanted parts, fractioning, etc.). This group includes cereals, roots and tubers, fruits, vegetables, meat and others. Processed culinary ingredients are substances extracted from natural foods or from nature and consumed as culinary preparation items, such as vegetable oil, lard, butter, sugar and salt. This study includes the unprocessed or minimally processed foods and processed culinary ingredients, in the same group, composed by 25 subgroups: rice, beans, other legumes, fresh fruits and juice, vegetables, roots and tubers, homemade cakes, corn and other cereal dishes, pasta, beef, pork, poultry, other meat, fish, seafood, eggs, typical Brazilian dishes, viscera, milk, yogurt and natural curd, coffee and tea, nuts and seeds, butter, oil and other culinary ingredients.

Processed food consists of manufactured products prepared essentially by adding processed culinary ingredients (such as salt, sugar and oil) to unprocessed or minimally processed food in order to extend shelf life and improve palatability (such as canned and bottled vegetables or fruit). This group encompasses 6 subgroups: processed bread, processed cheese, canned vegetables, roots and tubers, processed meat, preserved fruits and fermented alcoholic beverages.

Ultra-processed foods are made mainly or solely of industrial ingredients and their production process involves complex manufacturing techniques used exclusively by industry. The purpose of its processing is to create durable, accessible, convenient, highly palatable and ready-to-consume food. This study divides this category into 15 subgroups: salty crackers and pastries, sweets, breakfast cereals, bakery products, ultra-processed bread, ultra-processed cheese, ultra-processed meat, margarine, sauces, ready-to-eat meals, soft drinks, artificial juices, dairy drinks, other ultra-processed beverages and distilled alcoholic beverages.

### 2.3. Statistical Analysis

Exploratory factor analysis was used to identify out-of-home eating patterns, through the correlation matrix applied to the 46 food subgroups. These subgroups were expressed in calories per capita obtained using the average of the two-day logs. Orthogonal rotation was applied to simplify the data structure [17,18]. The number of factors selected was chosen based on two strategies: eigenvalue >1 and the scree plot assessment [19,20]. Food subgroups with a factor loading greater than 0.30 or less than −0.30 were considered in the identification of each pattern [17]. Sample adequacy was checked using the Kaiser–Meyer–Olkin (KMO) test. The KMO can assume values between 0 and 1. Low values mean that the variables have too little in common to proceed with the analysis. Values below 0.5 are considered unacceptable [21]. In the present study, we obtained a KMO = 0.68.

Pattern scores were predicted for each sample individual. These values represent the adhesion to each pattern and were used as independent variables in the regression analysis.

The average score of each dietary pattern and 95% confidence intervals of the respective scores were described in the population according to sociodemographic characteristics, such as age (years), sex (male/female), residence area (urban/rural), regions (North, Northeast, Centre-West, South and Southeast), education level (up to 4, 5–8, 9–12, over 12 years), income quintiles.

Diet quality was evaluated by using the following markers: energy density excluding beverages (kcal/g), percentage of calories from macronutrients (carbohydrates, proteins, lipids, saturated fat and *trans* fat), percentage of calories from free sugar, fiber, calcium, iron, potassium and sodium densities (expressed in g or mg per 1000 kcal). All markers were estimated using the average of the total diet (including food consumed whether at home or out).

Linear regression models adjusted and standardized were used to estimate the predicted values of the diet markers and to test the association to the quintile of patterns’ scores. Adjustment took into account the same sociodemographic characteristics previous described, plus intensity of food eaten out (estimated by the percentage of calories consumed out of home in the total diet). The nutritional markers were the dependent variables and quintiles of each patterns’ scores were independent variables.

The significance level of 0.05 was adopted for all of this study’s analyses and sample weighting factors allowing for the extrapolation of the results to the Brazilian population were used. All analyses were performed using Stata 14 statistical software (StataCorp, College Station, TX, USA).

## 3. Results

Based on exploratory factor analysis, we devised three patterns of out-of-home eating (Table 1). These patterns explained 13.6% of variance. The first pattern carried more rice, beans, beef, poultry, pasta, roots and tubers, vegetables and eggs, called “traditional meal” because it is made according to typical Brazilian preparations. The second pattern called “typical Brazilian breakfast/tea” carried more processed bread, butter, margarine, milk, coffee and tea and processed cheese. The third pattern is exclusively composed of ultra-processed foods, such as soft drinks, ready-to-eat meals (fast food, appetizers, pizza, etc.) and sweets, which is called “ultra-processed food.”

Table 2 shows the average of each food pattern score according to sociodemographic characteristics. Teenagers and women are the groups that presented the lower average score for the “traditional meal” pattern. The highest income quintile presented higher average score for the “traditional meal” pattern when compared to the lowest quintiles.

The average score predicted for the “typical Brazilian breakfast/tea” pattern was significantly higher in the urban area when compared to rural area.

The adhesion to the “ultra-processed food” pattern rises according to the increase in education and income level and the decrease in age. Individuals who finished middle school and those aged 10–19 and 20–39 years had more adhesion to this pattern. The Northeast had the lowest medium score compared to other regions.

**Figure 1:**
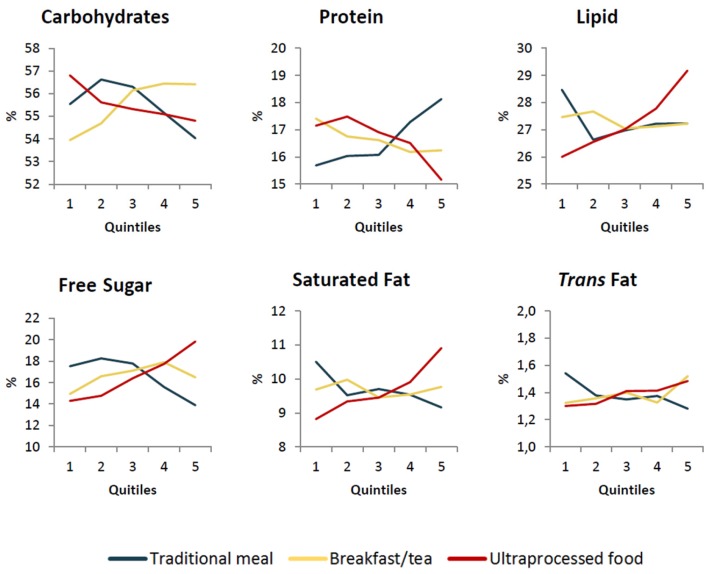
Predicted values of carbohydrates, protein, lipid, free sugar, saturated fat and *trans* fat percentage according to fifths participation in eating out pattern scores, adjusted for personal, sociodemographic and socioeconomic characteristics and the percentage of calories consumed outside of the home. Brazilian population aged 10 and up (2008–2009).

**Figure 2:**
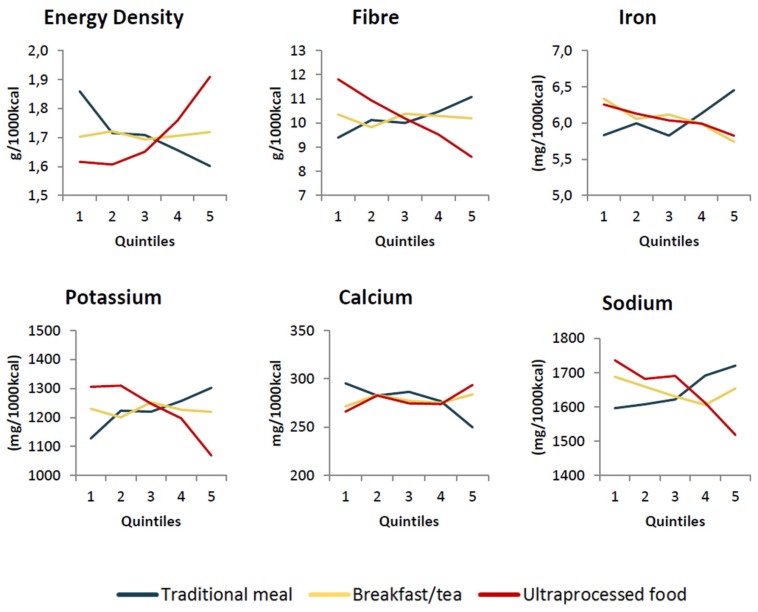
Predicted amount of energy density, fiber, iron, potassium, calcium and sodium content according to fifths participation in eating out pattern scores, adjusted for personal, sociodemographic and socioeconomic characteristics and the percentage of calories consumed out. Brazilian population 10 years old and over (2008–2009).

Figure 1 and Figure 2 show the adjusted predicted values of the nutritional indicators according to the quintiles of adhesion to each out-of-home eating pattern, while Table 3 presents the coefficients of association between the variables. It was possible to verify a negative association between the “traditional meal” pattern and energy density, the percentage of calories from carbohydrates, saturated fat, *trans* fat, free sugar and calcium. On the other hand, the percentage of calories from proteins, fiber, iron, potassium and sodium densities, were directly associated with the “traditional meal” pattern. The “typical Brazilian breakfast/tea” pattern was positively associated with the percentage of calories from carbohydrates, *trans* fat and free sugar and was negatively associated with the percentage of calories from protein, concentration of iron and sodium. The “ultra-processed food” pattern showed a positive association with the energy density, the percentage of calories from lipids, saturated fat, *trans* fat, free sugar and density of calcium and a negative association with the percentage of calories from carbohydrates and protein and from fiber, iron, potassium and sodium densities. Other associations were not significant.

## 4. Discussion

Our results support the existence of three different patterns of out-of-home eating among Brazilian population and add information to previous literature suggesting that a healthy diet pattern out-of-home is feasible in a middle-income country. Generally, the “traditional meal” pattern presented a positive association with healthy nutritional indicators and a negative association with unhealthy nutritional indicators and it was dietary pattern which explained most of the variance of food eaten out-of-home in Brazil.

The “Typical Brazilian breakfast/tea” and “ultra-processed food” patterns, on the other hand, presented a positive association to unhealthy nutritional indicators, such as saturated fat and free sugar and negative association to healthy nutrients, such as iron.

Although no other research study has assessed patterns of eating out in Brazil, the results found are in line with other literature findings about dietary patterns and its association to diet quality. A study using food acquisition data in Brazil reported two patterns of consumption: a “mixed” pattern, including healthy items (such as fruits and vegetables) and unhealthy items (such as sweet, soft drinks and ready-to-eat meals) and a “traditional” pattern, composed mainly of rice and beans. As in this article, the “traditional” pattern showed a positive association with healthy nutrients, such as fiber, while the “mixed” pattern had a positive association with unhealthy markers such as lipids, saturated fat and addition sugar [22].

Diets with excessive intake of saturated fat and *trans* fat increase the risk of development of NCDs [23,24], while diets with high energy density [25] and high percentage of free sugar [26] increase the risk of weight gain. The excessive consumption of free sugar is also related to the increase of dental cavities and the risk of obesity [26,27].

“Ultra-processed food” pattern also shows a negative association to fiber, potassium and iron content. Low fiber consumption is associated with the development of obesity, diabetes, cardiovascular diseases and different types of cancer [1,28], while an insufficient potassium intake is a risk factor for arterial hypertension [29]. Iron deficiency has a number of consequences, most notably child growth delay and the increase of fetal and maternal mortality [30].

Studies demonstrated that diets with higher protein contents, which were positively associated with “traditional meal” patterns, are related to the increase of dietary thermogenic and satiety, when compared to lower contents of this nutrient. Therefore, a higher protein participation may contribute to weight loss [31]. On the other hand, the “traditional meal” pattern was positively associated with sodium concentration. The excessive intake of this nutrient is related to increased blood pressure [1,32,33], development of cardiovascular diseases and stroke [34,35]. This association is probably a reflection of Brazilian culture, as it is common practice to use large amounts of salt in culinary preparations [36].

The calcium concentration (essential nutrient in neuromuscular function, blood coagulation and bone stiffness) was negatively associated with “traditional meal” pattern, which is possibly related to the absence of this nutrient in food sources (such as milk) in the pattern. The positive association between “ultra-processed food” and calcium content, on the other hand, can be explained by ultra-processed food item sources of this nutrient, such as ready-to-eat meal and fast-food, which usually contain cheese as a culinary ingredient [37].

Considering that “ultra-processed food” pattern is exclusively composed of ultra-processed food, the results found in the present study, similarly to other articles, show an association between this food group consumption and the increase of lipids, saturated fat, trans fat, free sugar and decrease of fiber, iron and potassium content [37,38].

In addition to identifying eaten out patterns and its impact on diet quality, the present paper also identified vulnerable groups adherence to unhealthy diet patterns. Women, for example, showed higher average score for “ultraprocessed food” pattern when compared to “traditional meal” patterns. This result is in line with another study carried out in Brazil that points out the higher consumption of ultraprocessed foods among women when compared to men [39]. This study suggested that different stress coping mechanisms between both sexes could be considered as a possible cause of the differences between men and women diets, since women are more susceptible to stress and it interferes directly in diet quality [40].

The fact that adolescents (10–19) represent the group with lower “traditional meal” pattern adhesion and higher “ultra-processed food” adhesion is concerning, as dietary habits developed during this period tend to be carried into adulthood [41,42]. Given that adolescents spend a considerable part of the day at school [43], this environment can be considered an important determinant in food consumption outside of the home for this age group. Although the Brazilian School Feeding Program promotes access to high quality food in public schools, the same does not necessarily occur in private schools. In addition, the sale of unhealthy foods and sugary beverages is common in commercial spaces inside or around schools, such as snack bars. These sales are associated with an increase in ultra-processed food consumption, suggesting the need for implementing laws to regulate commerce in these locations [44].

The influence of income and education level on eating out patterns also deserves attention. Our results showed that the “traditional meal” and “ultra-processed food” patterns adhesion increased according to income and education, which may seem contradictory but it is not, since these two patterns may represent the consumption in different meals outside home. The income is directly associated with the outside home consumption, as reported in Brazil [9] and in other countries [42]. Thus, the fact that the higher income class present greater adherence to both eaten out patterns, can be explained by the fact that such individuals made more than one meal out and presented different eating patterns in each occasion. Previous analyses carried out by our research group (unpublished data) show that high income people tend to eat more traditional meal out at lunch and ultra-processed foods at dinner.

In spite of our results, low socioeconomic status influences a low quality of meals eating out according to the literature [10,42]. In Brazil, a national study found that the cost of eating out for one week was higher in the traditional meals group (R$21.56) when compared to expenses associated with snacks groups such as soft drinks, fried appetizers and sweets (R$3.17, R$2.86 and R$2.02, respectively). In addition, the sweets group was the one with greatest consumption among lower income individuals, attributable to the low cost of “less healthy” food acquisition out-of-home [10].

In this sense, it is necessary to improve access to quality food through the creation of institutional restaurants or affordable self-service restaurants, for example [45].

In addition to the measures influencing adhesion to a healthy eating out pattern, other strategies can be used to discourage the adhesion to unhealthy eating out patterns. Strategies such as limiting ultra-processed food outlets, especially at workplaces and schools and regulation of food publicity, are important strategies to promote a healthy environment [46].

In Brazil, federal guidelines recommend restriction of sales and promotion of foods high in fat, saturated fat, trans fat, free sugar and salt in school environments, however the lack of a national law forbidding these foods, results in an unhealthy food environment in many Brazilian schools [47].

In regard to worker’s health, a promising development is the advent of the Promotion of Adequate and Healthy Food in Work Environments, published in ordinance No. 1.274 by the Ministry of Health (MH) on 7 July 2016. According to these guidelines, establishments located within the premises, as well as contractors for the supply of meals at events in MH and related entities should offer only unprocessed or minimally processed foods and homemade dishes. It also prohibits the direct sale, promotion and advertising of ultra-processed foods with excessive amounts of sugar, fat and sodium and limits the supply of processed foods [48]. This policy promotes adhesion to healthy eating out habits and should be expanded to other institutions, including other public institutions, hospitals and educational centers.

### Limitations and Strengths

It is important to emphasize that the present study has limitations related to the instrument used to collect information about food, such as underestimation of food consumption and modification of habitual consumption on the days of the study. In addition, food logging does not always provide sufficient information to distinguish homemade dishes from ultra-processed foods. To minimize some of this deviation, both days of the food log were used, the collection instrument was pre-tested and validated, quality control procedures were performed during data collection and inconsistent records were excluded and replaced with imputed values [12]. When information from the food log was insufficient to classify food according to NOVA, we assumed that the most common preparation use was the case, for example, hamburger and pizza that were considered as ultraprocessed foods.

POF’s out-of-home food definition does not consider food that has been prepared out and consumed at home, or food that has been prepared at home and consumed out, which may underestimate the prevalence of eating out but such obliquity does not interfere with the patterns identified.

Although no actual cooking recipes were used to estimate the nutritional composition of preparations, the table of nutritional composition used was constructed specifically for the POF 2008/2009, based on preparations closest to Brazilian habits. In addition, the probabilistic character of the sample studied allowed us to evaluate this study as being representative of adolescents and adults in Brazil.

Exploratory factor analysis has its limitations when applied to nutritional epidemiology studies; as a consequence of the great heterogeneity of food banks, the inter-correlation between variables is generally modest, which explains the low value found in the cumulative variance (13.6%) [49]. In addition, exploratory factor analysis was performed using a large number of variables, which interferes in the variance but increases the possibility of combinations and, consequently, the number of factors retained. Conversely, the patterns identified are consistent with the country’s eating habits, thus confirming the study’s validity.

It is also important to emphasize that this study was a pioneer on the identification of eating out patterns in Brazil using a representative sample of the population. Furthermore, the present paper demystifies the premise that eating out has necessarily a negative impact on the diet quality.

## 5. Conclusions

As demonstrated previously, the results indicate that dining out in Brazil may have a negative impact on diet when based on typical Brazilian breakfast/tea or ultra-processed food. But it is also possible to maintain a healthy diet out-of-home when adhering to traditional Brazilian cooking standards.

The “traditional meal” pattern was the first pattern retained in our model and explained most of the variance in foods eaten out-of-home. This is an important result, because shows that among Brazilian population, the minimally processed food pattern is predominant even out of home. Similar results were observed in Southern Europe, where restaurants were the principal source of out-of-home eating [50]. In the United States, on the other hand, fast food was the local that most contributed to out-of-home consumption [3].

Considering eating out an increasingly habit and the increasing prevalence of overweight and obesity, it is necessary to implement actions and strategies that aimed the promotion of healthy food choices and also the creation of environments that provide such choices.

## Figures and Tables

**Table 1:** Out-of-home eating patterns. Brazilian Population 10 years old and over (2008–2009).

	Traditional Meals ^a^	Typical Brazilian Breakfast/Tea ^b^	Ultra-Processed Food ^c^
Rice	0.81		
Beans	0.75		
Vegetables and greens	0.30		
Roots and tubers	0.45		
Pasta	0.41		
Beef	0.63		
Poultry	0.48		
Eggs	0.31		
Butter		0.51	
Milk		0.54	
Coffee and tea		0.36	
Processed bread		0.81	
Processed cheese		0.35	
Margarine		0.53	
Sweet			0.32
Ready-to-eat meals			0.66
Soft drinks			0.71

Only the food subgroups that present factor loadings superior to |0.30| were shown in the table. ^a^—composition: rice, beans, vegetables and greens, roots and tubers, pasta, beef, poultry, eggs; ^b^—composition: butter, milk, coffee and tea, processed bread, processed cheese, margarine; ^c^—composition: sweets, ready-to-eat meals, soft drinks.

**Table 2:** The score patterns average for meals eaten outside of the home according to personal, demographic and socioeconomic characteristics. Brazilian population aged 10 and up (2008–2009).

	Traditional Meals ^a^			Typical Brazilian Breakfast/Tea ^b^			Ultra-Processed Food ^c^		
	Average	95% CI *		Average	95% CI *		Average	95% CI *	
Brazil	0.01	−0.02	0.03	0.04	0.02	0.07	0.08	0.05	0.10
Age									
10–19	−0.32	−0.36	−0.29	−0.12	−0.15	−0.08	0.21	0.15	0.27
20–39	0.16	0.12	0.20	0.13	0.08	0.18	0.13	0.09	0.18
40–59	0.09	0.04	0.14	0.07	0.02	0.13	−0.09	−0.14	−0.02
60–over	−0.05	−0.13	0.06	−0.06	−0.17	0.05	−0.30	−0.37	−0.22
Sex									
Male	0.15	0.12	0.19	0.06	0.02	0.10	0.11	0.06	0.15
Female	−0.15	−0.18	−0.13	0.02	−0.01	0.06	0.04	0.00	0.08
Area									
Rural	0.00	−0.05	0.05	−0.04	−0.09	0.01	−0.27	−0.31	−0.23
Urban	0.01	−0.02	0.03	0.05	0.02	0.08	0.12	0.09	0.16
Education									
Up to 4	−0.01	−0.06	0.04	0.01	−0.04	0.07	−0.21	−0.25	−0.17
5–8	−0.09	−0.13	−0.04	0.06	0.00	0.11	0.03	−0.03	0.09
9–12	0.07	0.02	0.11	0.08	0.03	0.1 3	0.21	0.15	0.26
Over 12	0.06	0.00	0.12	−0.02	−0.08	0.04	0.26	0.18	0.33
Income									
Q1	−0.06	−0.10	−0.01	−0.05	−0.09	0.00	−0.24	−0,28	−0.19
Q2	−0.04	−0.09	0.00	0.03	−0.02	0.09	−0.05	−0.11	0.02
Q3	−0.02	−0.09	0.03	0.08	0.02	0.15	0.01	−0.04	0.06
Q4	0.05	0.00	0.11	0.11	0.04	0.17	0.17	0.10	0.24
Q5	0.07	0.02	0.12	0.02	−0.04	0.08	0.33	0.26	0.40
Region									
North	0.08	0.02	0.14	−0.04	−0.09	0.02	0.00	−0.05	0.06
Northeast	0.00	−0.04	0.04	0.01	−0.04	0.05	−0.11	−0.15	−0.07
Southeast	−0.01	−0.05	0.03	0.10	0.05	0.15	0.12	0.07	0.17
South	−0.01	−0.06	0.05	−0.01	−0.06	0.04	0.23	0.16	0.30
Centre-west	0.09	0.03	0.15	−0.04	−0.09	0.02	0.16	0.05	0.27

*—95% CI; CI—Confidence Interval. ^a^—composition: rice, beans, vegetables and greens, roots and tubers, pasta, beef, poultry, eggs; ^b^—composition: butter, milk, coffee and tea, processed bread, processed cheese, margarine; ^c^—composition: sweet, ready-to-eat meals, soft drinks.

**Table 3:** Association between dietary markers and fifths participation in each pattern scores adjusted for personal, sociodemographic and socioeconomic characteristics and the percentage of calories consumed outside of the home. Brazilian population aged 10 and up (2008–2009).

	Traditional Meals ^a^		Typical Brazilian Breakfast/Tea ^b^		Ultra-Processed Food ^c^	
	Coef. **	*p*	Coef. **	*p*	Coef. **	*p*
Energy density (g/1000 kcal)	−0.23	0.000 *	0.01	0.687	0.26	0.000 *
% carbohydrates	−0.05	0.002 *	0.11	0.000 *	−0.08	0.000 *
% protein	0.17	0.000 *	−0.09	0.000 *	−0.14	0.000 *
% lipids	−0.07	0.000 *	−0.02	0.097	0.16	0.000 *
% saturated fat	−0.14	0.000 *	−0.01	0.416	0.20	0.000 *
% *trans* fat	−0.09	0.000 *	0.05	0.000 *	0.07	0.000 *
% free sugar	−0.14	0.000 *	0.08	0.000 *	0.23	0.000 *
Fiber (g/1000 kcal)	0.14	0.000 *	0.00	0.712	−0.29	0.000 *
Calcium (mg/1000 kcal)	−0.10	0.000 *	0.02	0.186	0.05	0.000 *
Iron (mg/1000 kcal)	0.10	0.000 *	−0.10	0.000 *	−0.08	0.000 *
Potassium (mg/1000 kcal)	0.19	0.000 *	0.00	0.864	−0.26	0.000 *
Sodium (mg/1000 kcal)	0.10	0.000 *	−0.04	0.003 *	−0.16	0.000 *

* Significance values. ** Coefficients were adjusted and standardized for personal, sociodemographic and socioeconomic characteristics and the percentage of calories consumed outside of the home; ^a^—composition: rice, beans, vegetables and greens, roots and tubers, pasta, beef, poultry, eggs; ^b^—composition: butter, milk, coffee and tea, processed bread, processed cheese, margarine; ^c^—composition: sweet, ready-to-eat meals, soft drinks.
